# Vitamin B12 Deficiency Presenting as Pseudothrombotic Microangiopathy and Intestinal Pseudo-Obstruction

**DOI:** 10.7759/cureus.104246

**Published:** 2026-02-25

**Authors:** Erandy A Salcedo Elguea, Humberto Barrio Rivera, Pablo A Garcia Chavez

**Affiliations:** 1 Internal Medicine, Institute for Social Security and Services for State Workers General Hospital, Juarez City, MEX; 2 Surgery, Mexican Social Security Institute Hospital of Zone Number 35, Juarez City, MEX; 3 Hematology, Institute for Social Security and Services for State Workers General Hospital, Juarez City, MEX

**Keywords:** ineffective erythropoiesis, intestinal pseudo-obstruction, megaloblastic anemia, pernicious anemia, vitamin b12 deficiency anemia

## Abstract

Vitamin B12 deficiency is a common and reversible cause of megaloblastic anemia, but in severe cases, it may mimic thrombotic microangiopathy (TMA), a presentation known as pseudothrombotic microangiopathy (pseudo-TMA). This rare entity is frequently underrecognized and may lead to inappropriate therapies if not promptly identified. We report the case of a 62-year-old man who presented with abdominal pain, distension, and constipation, initially raising concern for intestinal obstruction or malignancy. Laboratory evaluation revealed macrocytic anemia, thrombocytopenia, elevated lactate dehydrogenase, and indirect hyperbilirubinemia. Further assessment demonstrated a low reticulocyte count, absence of significant schistocytosis, markedly reduced vitamin B12 levels, and elevated ferritin. Autoimmune testing confirmed pernicious anemia as the underlying etiology. Treatment with intramuscular hydroxocobalamin resulted in complete clinical and hematologic recovery. This case demonstrates the value of recognizing pseudo-TMA and considering vitamin B12 deficiency in patients with TMA-like features and unexplained gastrointestinal symptoms.

## Introduction

Vitamin B12 deficiency represents an important and potentially reversible cause of macrocytic anemia and systemic dysfunction [1]. In severe cases, it may present with laboratory findings that closely resemble thrombotic microangiopathy (TMA), including cytopenias, elevated lactate dehydrogenase levels, indirect hyperbilirubinemia, and schistocytosis on peripheral smear. In fact, a systematic review by Tran and Tran reported approximately 76% of patients with cobalamin deficiency-related pseudo-TMA exhibited schistocytes on peripheral blood smears [2]. This constellation of findings, referred to as pseudo-TMA, can closely resemble true TMA syndromes and may lead to diagnostic uncertainty [2-4]. Although clinical and laboratory features often guide the initial evaluation, additional studies, such as ADAMTS13 activity, may assist in distinguishing true thrombotic thrombocytopenic purpura (TTP) from mimicking conditions when clinically indicated [5]. Failure to recognize this entity can lead to misdiagnosis and expose patients to unnecessary interventions such as plasma exchange or immunosuppressive therapy [2,3,6,7].

Pseudo-TMA and true TMA differ in several clinically relevant aspects. Pseudo-TMA more often affects older individuals and typically follows a subacute course, evolving over weeks to months with nonspecific symptoms, such as fatigue and pallor, and neurologic manifestations such as paresthesias or gait instability [2]. In contrast, true TMA syndromes, including TTP and hemolytic uremic syndrome (HUS), generally present abruptly and may occur across a broader age range, often with more acute and severe clinical manifestations such as petechiae, purpura, altered mental status, renal impairment, and, in severe cases, fever or hemodynamic instability [5].

From a laboratory standpoint, vitamin B12 deficiency frequently produces hematologic abnormalities that may involve multiple cell lines. Macrocytic anemia is present in approximately 55% of cases, thrombocytopenia in 10%, and leukopenia in about 14% [8]. In the subset of patients who develop pseudo-TMA, findings typically include macrocytic anemia, mild to moderate thrombocytopenia, low reticulocyte counts, markedly elevated LDH levels disproportionate to the degree of anemia, and limited schistocytosis on peripheral smear [3,4]. By comparison, true TMA is characterized by normocytic anemia, severe thrombocytopenia, reticulocytosis, LDH elevation consistent with active hemolysis, and prominent schistocytosis [5].

Pernicious anemia is one of the most common causes of severe vitamin B12 deficiency in adults. It results from autoimmune-mediated destruction of gastric parietal cells, leading to intrinsic factor deficiency and impaired cobalamin absorption [1]. Although hematologic abnormalities are often the predominant clinical manifestation, vitamin B12 deficiency may also affect multiple organ systems, particularly the nervous system.

Vitamin B12 plays a critical role in DNA synthesis and normal cellular replication. Its deficiency can cause neurological complications through impaired myelin synthesis, the accumulation of methylmalonic acid, and hyperhomocysteinemia [9,10]. While classic manifestations involve the central and peripheral nervous systems, dysfunction of the autonomic and enteric nervous systems has also been described, potentially leading to gastrointestinal motility disorders. Clinical presentations, such as gastroparesis or intestinal pseudo-obstruction, have been reported in association with vitamin B12 deficiency, even in the absence of mechanical obstruction [11,12]. However, the evidence supporting a direct causal relationship in patients without other underlying comorbidities remains limited and is largely based on case reports and small observational studies. These factors may further complicate the diagnostic process, particularly when hematologic abnormalities suggestive of TMA are present.

Here, we present a case of pseudothrombotic microangiopathy secondary to pernicious anemia with an unusual abdominal presentation that mimics intestinal pseudo-obstruction. This report aims to highlight the diagnostic challenges associated with severe vitamin B12 deficiency and to emphasize the importance of considering this reversible condition in patients presenting with laboratory findings suggestive of thrombotic microangiopathy and unexplained gastrointestinal symptoms.

## Case presentation

A 62-year-old male with a history of cholecystectomy and inguinal hernioplasty performed 10 years prior to admission presented to the emergency room with a 4-day history of progressive abdominal pain. He reported no other significant past medical history and was not taking any medications. He denied dietary restrictions and described maintaining a balanced diet. He reported occasional alcohol consumption, approximately one to two drinks every four months. He denied unintentional weight loss.

The abdominal pain was localized to the mesogastrium, described as cramping, non-radiating, and rated as 8/10 in intensity, with worsening after meals. He also reported abdominal distension, nausea without vomiting, general fatigue, and absence of bowel movements for five days prior to presentation. On directed questioning, he additionally described progressive weakness and intermittent paresthesias in the lower extremities over the preceding six months.

On arrival, the patient was hemodynamically stable. A physical exam showed a distended abdomen with decreased bowel sounds, generalized tympany on percussion, and tenderness to both superficial and deep palpation in the mesogastric region, without guarding or signs of peritoneal irritation. No glossitis, ataxia, or focal neurological deficits were observed.

Initial abdominal X-ray revealed the absence of rectal gas, raising concern for an intestinal obstruction. Laboratory evaluation revealed macrocytic anemia and mild thrombocytopenia, along with biochemical markers consistent with hemolysis, including elevated lactate dehydrogenase and indirect hyperbilirubinemia (Table 1). Given the persistent constipation and the elevated lactate dehydrogenase level, colorectal malignancy was initially considered in the differential diagnosis. Contrast-enhanced abdominal computed tomography and tumor marker testing were performed afterward, both showing no signs of mechanical obstruction or neoplastic disease.

**Table 1:** Laboratory data during hospitalization and follow-up MCV: mean corpuscular volume; LDH: lactate dehydrogenase

	Reference values	09/07/2025	18/07/2025	21/07/2025	21/08/2025
Hgb (g/dL)	13-18	8.8	7.3	-	15.5
MCV (fL)	80-100	106.7	103.6	-	90.20
Platelets (10^3^μl)	150-400	124	99	-	229
Leukocytes (10^3^μl)	3-15	6.88	3.96	-	13.10
LDH (U/L)	135-225	1797	1550	-	240
Total bilirubin (mg/dL)	0.2-1.30	2.0	1.9	-	0.7
Unconjugated bilirubin (mg/dL)	0-1.0	1.1	1.1	-	0.3
Aspartate aminotransferase (AST)	15-55	46	45	-	21
Creatinine (mg/dL)	0.7-1.5	1.1	0.9	-	0.99
Ferritin (ng/mL)	-	-	-	897	24

**Figure 1:**
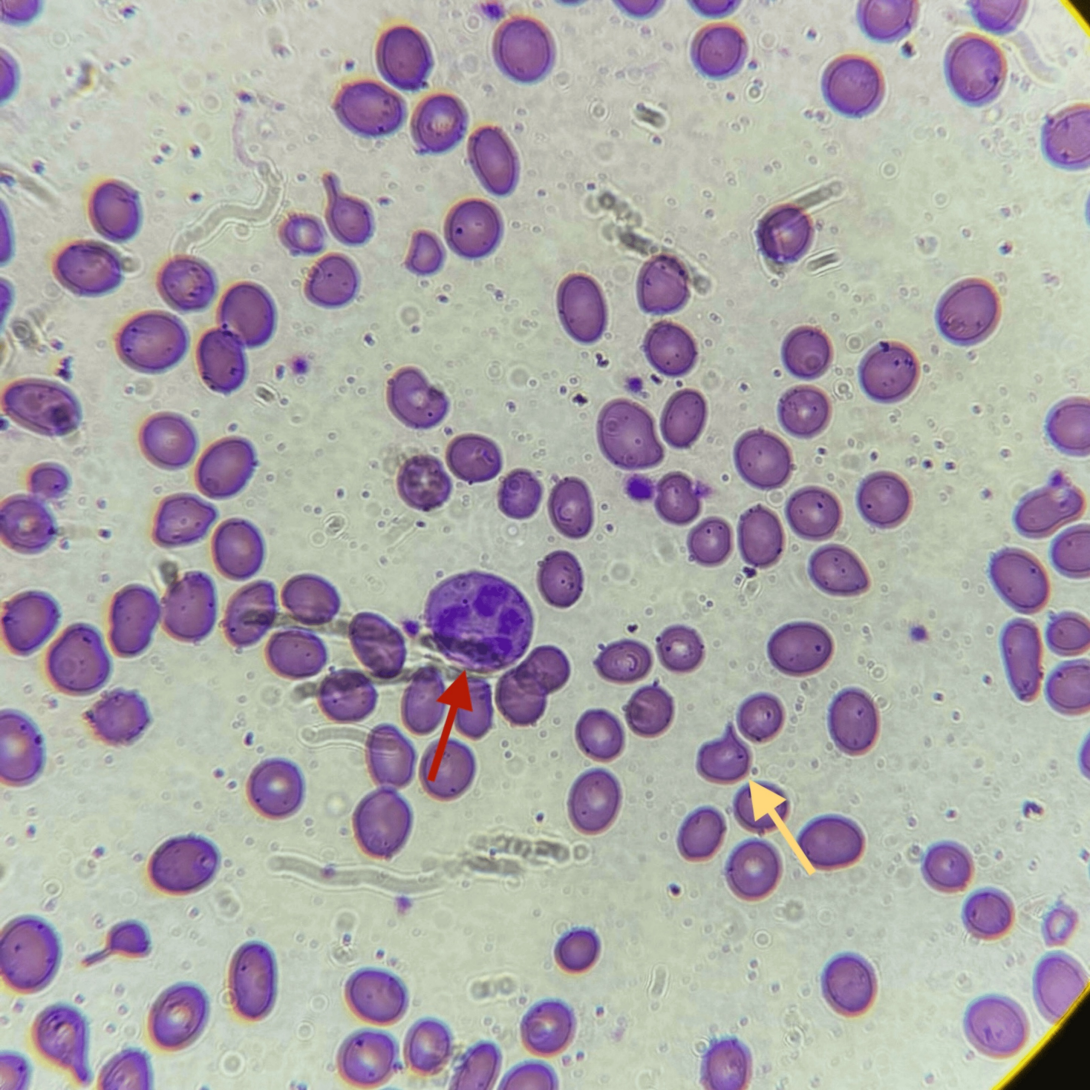
Peripheral blood smear findings Peripheral blood smear shows anisocytosis with multiple ovalocytes. Hypersegmented neutrophils are observed (red arrow), along with rare schistocytes (yellow arrow).

Due to persistent anemia during hospitalization, a hematology consultation was obtained. Further evaluation demonstrated a low reticulocyte count. Peripheral blood smear examination showed hypersegmented neutrophils, anisocytosis with numerous ovalocytes, and few schistocytes (Figure 1).

Additional laboratory testing revealed a markedly reduced serum vitamin B12 level of 100 pg/mL, elevated ferritin levels, and normal folate concentrations (Table 1). A direct antiglobulin (Coombs) test was negative. Haptoglobin levels, ADAMTS13 activity, methylmalonic acid, and homocysteine measurements were not available due to institutional resource limitations. Collectively, these findings supported a diagnosis of vitamin B12 deficiency with ineffective erythropoiesis and secondary biochemical evidence of hemolysis.

To further investigate the etiology of the vitamin B12 deficiency, autoimmune testing was performed. Antiparietal cell antibodies were positive, while anti-intrinsic factor antibodies were negative, supporting a diagnosis of pernicious anemia.

The patient was administered intramuscular hydroxocobalamin at a dose of 1,000 µg daily for five days, then received weekly injections, followed by monthly maintenance therapy. No blood transfusions or surgeries were needed. The patient tolerated the treatment well and showed gradual clinical improvement.

At the six-week follow-up, laboratory studies showed complete resolution of anemia, normal red blood cell indices, and the disappearance of biochemical markers of hemolysis, including lactate dehydrogenase and indirect bilirubin. Clinically, the patient reported complete resolution of abdominal pain and distension, return of normal bowel movements, and no remaining symptoms. He remained adherent to vitamin B12 supplementation and experienced no complications.

## Discussion

Pseudo-TMA is a rare but well-recognized manifestation of severe vitamin B12 deficiency, accounting for approximately 2-3% of patients with profound cobalamin deficiency [8,13]. Because it closely mimics true thrombotic microangiopathies, such as TTP, this condition is often underrecognized, leading to delays in diagnosis and potentially inappropriate use of plasma exchange or immunosuppressive therapies [2,3,6,7].

Although both entities share anemia, thrombocytopenia, and biochemical evidence of hemolysis, important pathophysiologic and laboratory differences exist. True TMA is characterized by peripheral hemolysis caused by the mechanical fragmentation of erythrocytes within the microvasculature, typically due to severe ADAMTS13 deficiency in TTP or endothelial injury in other TMAs [5,14]. This peripheral destruction leads to reticulocytosis, significant schistocytosis on peripheral smear, and moderate-to-marked elevations in lactate dehydrogenase (LDH).

In contrast, pseudo-TMA results from ineffective erythropoiesis due to impaired DNA synthesis in severe vitamin B12 deficiency. The primary mechanism is intramedullary hemolysis, in which erythroid precursors undergo apoptosis within the bone marrow before reaching the circulation [1,10]. This process produces laboratory findings that mimic hemolysis--elevated LDH and indirect bilirubin--but without true peripheral red cell fragmentation. Consequently, reticulocyte counts are typically low or inappropriately normal, reflecting marrow failure rather than a compensatory response [3,13].

Taking these differences into account, the assessment of ADAMTS13 activity plays a pivotal role in the diagnostic evaluation of suspected TTP. Severe ADAMTS13 deficiency strongly supports the diagnosis of true TMA, particularly TTP, whereas preserved activity favors alternative etiologies such as severe cobalamin deficiency [5,14]. When available, incorporation of ADAMTS13 testing into the diagnostic workup can therefore facilitate early differentiation between pseudo-TMA and true TMA and help avoid unnecessary plasma exchange.

Another key difference between pseudo-TMA and true TMA lines is in the peripheral blood smear findings. In pseudo-TMA, the smear typically includes macro-ovalocytes, anisocytosis, poikilocytosis, and hypersegmented neutrophils, reflecting megaloblastic hematopoiesis [1,10]. Although schistocytes may be present, they are usually less prominent and appear in the broader context of megaloblastic changes. In contrast, true TMA is characterized by more significant schistocytosis resulting from mechanical red cell fragmentation.

The degree of lactate dehydrogenase (LDH) elevation represents another useful distinguishing feature. In pseudo-TMA, LDH levels are often markedly elevated, frequently exceeding levels commonly observed in true TMA, sometimes reaching values above 2,500-3,000 IU/L [3,13]. This disproportionate elevation is attributed to the destruction of nucleated erythroid precursors in the marrow, which contain higher intracellular LDH concentrations than mature erythrocytes. In contrast, while LDH is elevated in true TMA due to peripheral hemolysis and tissue ischemia, the degree of elevation is often less extreme relative to the severity of anemia [3].

Similarly, iron parameters may provide additional diagnostic clues. In vitamin B12 deficiency, ineffective erythropoiesis leads to intramedullary destruction of erythroid precursors and iron trapping within the reticuloendothelial system, resulting in elevated ferritin levels, while true TMA usually does not present with hyperferritinemia unless inflammation is involved [10,15]. In this case, elevated ferritin levels likely reflected marrow iron sequestration caused by ineffective erythropoiesis.

Beyond hematologic involvement, vitamin B12 deficiency can affect the nervous system through impaired myelin synthesis, accumulation of methylmalonic acid, and hyperhomocysteinemia [10,11]. While classical manifestations involve the central and peripheral nervous systems, autonomic and enteric nervous system dysfunction may occur, potentially explaining gastrointestinal dysmotility and pseudo-obstruction in the absence of mechanical disease [11,12]. In our patient, abdominal symptoms were the initial presentation and contributed to diagnostic uncertainty, highlighting the multisystemic effects of severe vitamin B12 deficiency.

Regarding etiology, vitamin B12 deficiency can result from several causes, including inadequate dietary intake, malabsorption syndromes, gastrointestinal surgery, medication effects, and autoimmune disorders [10]. Among these, pernicious anemia is one of the most common causes of severe deficiency in adults and is characterized by an autoimmune destruction of gastric parietal cells, leading to a deficiency of intrinsic factor and impaired cobalamin absorption [1,10]. In our patient, positive antiparietal cell antibodies supported this diagnosis. However, antiparietal antibodies are not fully specific and do not definitively confirm pernicious anemia. Although anti-intrinsic factor antibodies are more specific, they were negative in this case. Based on these findings, upper endoscopy with gastric biopsy is planned to further evaluate for autoimmune gastritis [16].

This case has several limitations. ADAMTS13 activity testing was not available, which would have further strengthened the exclusion of TTP. Serum methylmalonic acid and homocysteine levels were not measured, limiting the biochemical confirmation of cobalamin deficiency. Haptoglobin levels were also unavailable, limiting further characterization of hemolysis. Furthermore, upper endoscopy with biopsy was not performed, preventing histologic confirmation of autoimmune gastritis despite the presence of antiparietal cell antibodies. Formal gastrointestinal motility studies were likewise not conducted, which could have provided objective documentation of enteric nervous system dysfunction. Despite these limitations, the rapid hematologic and clinical response to vitamin B12 therapy strongly supports the diagnosis of pseudo-TMA secondary to severe cobalamin deficiency. Table 2 lists the differences between pseudo-TMA and true TMA.

**Table 2:** Clinical and laboratory differences between pseudo-thrombotic microangiopathy and true thrombotic microangiopathy TMA: thrombotic microangiopathy; LDH: lactate dehydrogenase

Feature	Pseudo-TMA	True TMA
Type of hemolysis	Intramedullary	Intravascular/peripheral
Reticulocyte count	Low or normal	Elevated
Peripheral schistocytes	Few to moderate	Prominent
LDH	Markedly elevated, disproportionate to anemia	Elevated, proportional to hemolysis
Indirect bilirubin	Mild to moderate elevation	Elevated
Cell morphology	Macrocytosis, hypersegmented neutrophils	Normocytic, normal neutrophils
Ferritin	Elevated	Normal or mildly elevated
Platelets	Mild thrombocytopenia	Marked thrombocytopenia
Treatment	Vitamin B12 replacement	Plasma exchange, treat the underlying cause, and immunosuppression

## Conclusions

This case highlights pseudo-TMA as a rare but serious manifestation of severe vitamin B12 deficiency and emphasizes pernicious anemia as an important underlying cause. Differentiation from true TMA relies on recognizing key diagnostic features, such as intramedullary rather than peripheral hemolysis, low reticulocyte counts, limited schistocytosis, disproportionately elevated LDH levels, macrocytosis with hypersegmented neutrophils, elevated ferritin due to ineffective erythropoiesis, and, when available, assessment of ADAMTS13 activity.

Although gastrointestinal symptoms were prominent in this case, a direct causal relationship with enteric neuropathy cannot be definitively established and should be interpreted cautiously, given the limitations of the diagnostic workup. Early recognition of pseudo-TMA and its distinguishing features remains essential to avoid unnecessary plasma exchange and to ensure prompt recovery with appropriate cobalamin replacement.
